# Bilateral corneal pannus in Crohn's disease and assumed adalimumab-associated ocular surface disease

**DOI:** 10.1016/j.ajoc.2025.102424

**Published:** 2025-09-01

**Authors:** Nefeli Eleni Kounatidou, Johannes Birtel, Nicole Stuebiger

**Affiliations:** Department of Ophthalmology, University Medical Center Hamburg-Eppendorf, Hamburg, Germany

**Keywords:** Crohn's disease, Corneal pannus, Adalimumab, TNF-Alpha inhibitors, Ocular surface disease, Drug-induced complications

## Abstract

**Purpose:**

Anti-tumor necrosis factor (TNF)-alpha inhibitors are commonly used in the treatment of inflammatory bowel disease (IBD). Despite a beneficial risk-profile, dermatologic and ocular complications may particularly develop at higher doses. Here, we report a case of bilateral corneal pannus in a patient with Crohn's disease, which occurred following a prolonged adalimumab overdose.

**Observations:**

A 19-year-old male with Crohn's disease presented with progressive bilateral blurred vision, conjunctival injection, and light sensitivity. Clinical examination, including best-corrected visual acuity (BCVA), slit-lamp biomicroscopy, and funduscopy, revealed severe bilateral corneal pannus with vascularization extending superior to the limbus. The patient had been mistakenly administering adalimumab (40 mg) weekly instead of biweekly for over two years. Given the suspected medication-induced ocular surface disease, adalimumab was discontinued, and the α4β7 integrin antagonist vedolizumab was initiated. Adjunctive topical and systemic therapy was also implemented. At the 2-month follow-up, corneal pannus regressed, visual acuity improved, and ocular symptoms significantly decreased. Further improvement was observed at the 4-month follow-up.

**Conclusions and importance:**

Bilateral corneal pannus can be a rare ocular complication of long-term TNF-alpha inhibitor therapy. Particularly in young IBD patients, dermatologic and ocular complications should be considered. In severe cases, modification of the immunosuppressive agent along with targeted therapy should be implemented to prevent vision loss.

## Highlights

• Prolonged adalimumab overdose can cause severe ocular surface disease in IBD patients.
• Modification of the immunosuppressive agent and early intervention can improve ocular symptoms and vision.
• Monitoring for dermatologic and ocular side effects in young IBD patients on high-dose TNF-alpha inhibitors is essential.

## 1 Introduction

Anti-tumor necrosis factor (TNF)-alpha therapy is a cornerstone in the treatment of chronic immune-mediated diseases, such as inflammatory bowel disease (IBD).^1^ Although this therapy is generally well tolerated, dermatologic complications may occur or become exacerbated.^2^^,^^3^ Dermatologic diseases, such as rosacea, may manifest with higher prevalence in patients with IBD and recent studies suggest a causal relationship between the two conditions.^4^ Here, we report a case of facial dermatologic complications and bilateral corneal pannus in a patient with Crohn's disease under adalimumab (ADA) therapy.

## 2 Case report

A 19-year-old male patient with Crohn's disease (CD) presented with a 4-month history of progressive blurred vision, light sensitivity, and “redness” in both eyes. Previous treatments, including topical corticosteroid and antibiotic eye drops, provided minimal relief. The patient had been on 40 mg adalimumab weekly for over two years to manage his IBD. His CD was treated efficiently, however, the patient mistakenly administered 40 mg every week instead of every other week, as recommended by his gastroenterologist. Medical and ophthalmic history was otherwise unremarkable.

**Fig. 1:**
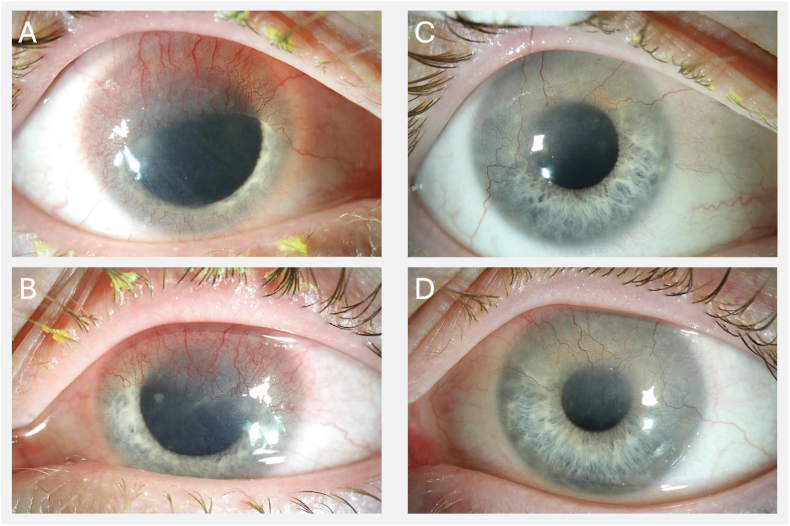
Slit-lamp photography at initial presentation (right column; A, right eye; B, left eye) showed bilateral conjunctival injection and a vascularized pannus extending from the upper limbus toward the optical axis. Follow-up examination after four months (C, right eye; D, left eye) demonstrated regression of the corneal vascularized fibrous network.

On examination, best-corrected visual acuity (BCVA) was 20/70 in the right and 20/100 in the left eye, respectively. Both eyes showed severe conjunctival injection and symmetrical corneal pannus, with vascularization extending from the superior limbus toward the optical axis (Fig. 1A and B). The anterior and posterior segment were otherwise unremarkable with mild meibomian gland dysfunction. Due to facial erythema, papules, and pustules on the cheeks, nose, and chin, a dermatological assessment was initiated and was suggestive of rosacea-like lesions.

Systemic therapy with slow-release doxycycline 40 mg once daily was initiated, along with topical cyclosporine 0.1 % two times a day, dexamethasone eye drops 0.1 % three times a day, artificial tears four times a day, and autologous serum eyedrops four times a day. Eyelid hygiene once daily was also recommended. Topical tetracyclines were not initiated, as tetracycline eye drops or ointments were not available; systemic slow-release doxycycline was prescribed due to the comparable efficacy in ocular rosacea and to address the patient's concurrent dermatologic findings.^5^ Autologous serum eyedrops were initiated due to their high content of growth factors and anti-inflammatory properties, supporting ocular surface recovery in severe ocular surface disease.^6^ Adalimumab was replaced by vedolizumab (α4β7 integrin antagonist) assuming that the corneal pannus might have been induced by an adalimumab overdose. At the 2-months follow-up, the corneal pannus was regressed, the patient reported reduced light sensitivity and BCVA improved to 20/50 in the right and 20/30 in the left eye, respectively; further improvement was noted at 4 months with BCVA improvement to 20/20 in the right and 20/25 in the left eye and residual corneal astigmatism of −1.9 dpt and −2.9 diopters on the right and left eye, respectively (Fig. 1C and D). A trial of rigid gas-permeable contact lenses was recommended, however, the patient declined that due to discomfort and foreign body sensation at initial fitting. Treatment with topical cyclosporine, autologous serum eye drops, and artificial tears was continued at the initial dosing frequency for maintenance purposes, while topical steroids were gradually tapered and eventually maintained at a dosage of once every other day.

## 3 Discussion

Dermatologic complications represent the most common adverse events of anti-TNF therapy, with higher incidence in IBD patients compared to other indications.^2^ These complications are particularly seen in younger patients and at higher doses.^2^ Additionally, epidemiologic studies have shown that IBD patients have a 2.1 higher risk of developing rosacea like diseases.^7^ The presented case highlights a potential link between ADA therapy – possibly aggravated by the mistakenly ADA overdosing - and exacerbation of rosacea leading to severe ocular surface disease in a CD patient.

There are indications supporting that the pannus development in our patient resulted from a severe blepharoconjunctivitis as a dermatologic complication of the adalimumab therapy. However, it is important to note that this link is indirect, as the facial rosacea presumably improved first after stopping ADA, which then resulted in secondary improvement of the ocular surface disease. The patient experienced improvement after discontinuing adalimumab, suggesting a possible link between the medication and ocular surface disease, including corneal pannus formation. Moreover, a weekly adalimumab administration and a young age (<28 years) at treatment initiation have been associated with increased risk of dermatologic complications, which could have led to severe bilateral blepharoconjunctivitis.^1^^,^^3^

This case highlights the need to recognize dermatologic and ocular complications from long-term TNF-alpha inhibitor treatment to prevent vision loss. In severe cases, topical treatment and modification of the immunosuppressive agent should be considered.^2^

## CRediT authorship contribution statement

**Nefeli Eleni Kounatidou:** Writing – original draft, Visualization, Data curation, Conceptualization. **Johannes Birtel:** Writing – review & editing, Supervision, Methodology. **Nicole Stuebiger:** Writing – review & editing, Supervision, Project administration, Methodology, Investigation, Data curation.

## Patient consent

Written consent to publish this case has not been obtained. This report does not contain any personal identifying information.

## Acknowledgements and disclosures

No funding or grant support was obtained.

## Authorship

All authors attest that they meet the current ICMJE criteria for Authorship.

## Declaration of competing interest

The authors declare that they have no known competing financial interests or personal relationships that could have appeared to influence the work reported in this paper.
